# Pre-movement EEG microstates reflect intended lifted load of volitional movement

**DOI:** 10.3389/fncom.2026.1784913

**Published:** 2026-03-23

**Authors:** Rohit Kumar Yadav, Sutirtha Ghosh, Lalan Kumar, Shubhendu Bhasin, Sitikantha Roy, Ratna Sharma, Suriya Prakash Muthukrishnan

**Affiliations:** 1 Department of Physiology, All India Institute of Medical Sciences, New Delhi, India; 2 Department of Electrical Engineering, Indian Institute of Technology Delhi, New Delhi, India; 3 Department of Applied Mechanics, Yardi School of Artificial Intelligence, Indian Institute of Technology Delhi, New Delhi, India

**Keywords:** Electroencephalography, load estimation, movement intention, movement planning, scalp electrical potential topography, volitional movement

## Abstract

**Introduction:**

Load estimation is one of the essential parameters for assistive robotic control in cases of rehabilitation. The high temporal resolution of the Electroencephalography (EEG) technique makes it the best tool to resolve the temporal dynamics of movement intention and planning. The quasi-stable scalp electrical potential topography represented by the EEG microstates could assess the real-time information processing in the brain for controlling assistive devices. We hypothesize that the EEG microstate preceding the movement could reflect the increasing load during a biceps curl movement.

**Methods:**

Ten healthy participants performed biceps curl movements, while their brain activity and muscle activation was recorded using EEG and EMG.

**Results:**

Eight microstate maps were found to represent the functional brain state before the movements. Two pre-movement microstate maps were found to reflect the load increments. The source maxima of these two reflective microstates maps were localized at the right insula and cingulate gyrus.

**Discussion:**

Our results imply that the load increments of volitional movement could be reflected by the pre-movement EEG microstates.

## Graphical Abstract

**Figure:**
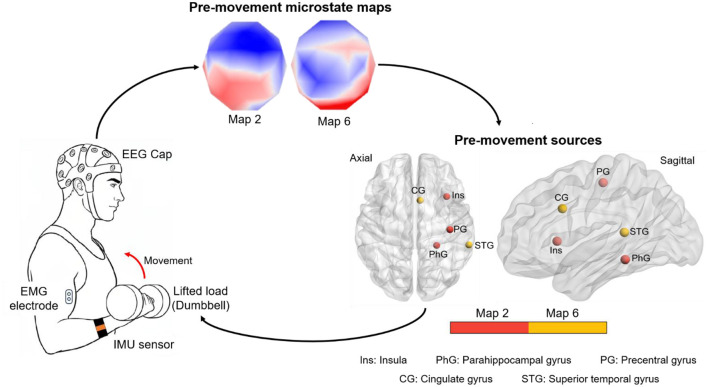

## 1 Introduction

Assistive control of peripheral muscles by biological signals and the feedback of the same is being researched on extensively. One of the challenges is predicting the load to be lifted by a person before the movement execution from neural signals is difficult. Does brain activity preceding a known movement contain information regarding the impending load to be encountered?

Accurate decoding of such information has strong implications for assistive control, neurorehabilitation, and Brain-Computer Interfaces (BCIs), enabling more responsive and adaptive user intent decoding. Motor BCIs aim to decode movement intentions from brain signals, which can be further combined with neurorehabilitative devices and assistive technology.

Understanding the brain activity preceding a movement that determines the kinetics (e.g., the load lifted) and kinematic parameters of the action to be executed has been a longstanding unresolved question in motor neuroscience. Humans are capable of performing a diverse range of movements, from simple mouse clicks to advanced body training activities such as dance or gymnastics (Liu and Ballard, 2021). These movements require coordination of the brain areas involved in sensorimotor prediction, sensory integration, movement planning, decision-making, movement execution, and performance evaluation (Gwin et al., 2011; Schwartz, 2016; Zhang et al., 2024). The precise timing between postural control and movement onset, with anticipatory postural adjustments, is a key factor contributing to balance and efficiency. These adjustments must be flexibly adapted to the specific properties of the object, such as its load or friction, which can change unexpectedly and are not always apparent to the individual before contact (Zhang et al., 2025).

While prior studies have decoded a variety of motor movements, including elbow extension/flexion, forearm pronation/supination, palmar/lateral grasp, hand opening/closing, palmar grasp, pincer grasp, lateral grasp, wrist supination, and pinch (Borràs et al., 2022; Hochberg et al., 2012; Mammone et al., 2020; Ofner et al., 2019; Schwarz et al., 2018, 2020a,b; Vuckovic and Sepulveda, 2008; Xu et al., 2021), less has been studied to decode load levels before movement onset.

The biceps curl, a single-joint elbow flexion movement, provides an ideal model for studying motor control due to its well-defined kinematics and minimal postural confounds (Jiang et al., 2025). Lifted loads during this movement offer a controlled paradigm to assess load-dependent neural changes in preparatory brain activity. While wearable rehabilitation devices can predict movement speed using motion encoders (Porciuncula et al., 2018), predicting load without relying on mechanical sensors remains an unresolved topic. Current exoskeletons or robotic prostheses rely on embedded mechanical sensors in gloves or cuffs, which add inertia, reduce comfort, and often fail to detect sudden load changes. Non-invasive neural correlates with intended effort could therefore replace or augment force sensors, enabling lighter, more intuitive controllers that adapt torque before movement begins (Gui et al., 2019; Zhang et al., 2024).

Assessing physiological signals such as electroencephalography (EEG), a cost-effective, non-invasive tool with high temporal resolution, has proven valuable for classifying lifted loads and differentiating between movement types (Qin et al., 2022). Distinct movements with unique kinetic profiles can be reliably detected from EEG patterns (Iturrate et al., 2018; Slobounov et al., 1999), and despite its limited spatial resolution, complex functional movements have been successfully decoded using advanced EEG signal processing techniques (Kaviri and Vinjamuri, 2025; Mohseni et al., 2020). Along with EEG, surface electromyography (EMG) has been studied to characterize neuromuscular activation patterns associated with movement execution and force generation (De Luca, 1997; Farina et al., 1985). EMG amplitude and temporal features alter with load and effort increments, serving as a potential marker of movement intensity and physical demand (Beck et al., 2019; Vigotsky et al., 2018). Moreover, EMG activity has been reported to precede the observed force production by approximately 20–150 ms. Thus, capturing the motor command transmission from the cortex to muscle (Norman and Komi, 1979; Zheng et al., 2022). Despite this temporal advantage, EMG remains limited in its ability to capture the preparatory neural states before the movement onset (Enoka and Duchateau, 2008).

Recent advances in EEG research, in contrast, have demonstrated that cortical activity not only reflects movement preparation but also encodes the anticipated intensity of upcoming motor actions. Neural correlates captured during resistance-based tasks, such as biceps curls, have been shown to differentiate between varying load levels and even predict kinematic parameters before movement initiation (Deniz et al., 2022; Maszczyk et al., 2019). While classical EEG markers, such as the readiness potential (RP), have been widely used to study motor preparation, they exhibit inconsistent reliability and limited predictive value for movement intention (Khalighinejad et al., 2018; Schurger et al., 2012). Consequently, recent investigations have shifted toward analyzing pre-movement oscillatory activity, which provides a more temporally precise representation of movement intention.

Prior work has shown that EEG signals encode distinct neural states corresponding to movement intensity, with higher loads eliciting broader cortical recruitment and increased inter-regional coupling (Olsen et al., 2021; Zhang et al., 2024). Event-related perturbations such as β (13–30 Hz) desynchronization over sensorimotor areas and α (8–13 Hz) suppression over parieto-occipital regions consistently precede movement onset (Kilavik et al., 2013; Pfurtscheller and Lopes Da Silva, 1999). Nevertheless, the majority of prior research has focused on binary load conditions, often neglecting the graded and potentially nonlinear cortical adaptations that accompany progressive loading. These oscillatory modulations emerge from a distributed preparatory network encompassing the supplementary motor area (SMA), pre-SMA, and primary motor cortex (M1), mediating context-dependent adjustments in corticospinal excitability before movement onset (Côté et al., 2020; Jacobs et al., 2009). EEG microstates have been shown to reflect large-scale cortical network dynamics underlying cognitive processing, including motor preparation, offering an ideal approach for characterizing rapid temporal transitions (Michel and Koenig, 2018).

EEG microstates represent brief, quasi-stable patterns of global scalp activity that reflect coordinated activation within large-scale neural networks (Lehmann et al., 1987). The temporal characteristics of microstates duration, frequency of occurrence, coverage, and global explained variance (GEV) quantify their stability and contribution to overall brain activity (Dinov and Leech, 2017; Khanna et al., 2015; Lehmann et al., 2009). These parameters capture the changes in cortical coordination independent of signal polarity, as polarity reversals do not alter the underlying spatial configuration (Dinov and Leech, 2017). Unlike conventional time- or frequency-domain analyses, microstate analysis captures the quasi-stable sequential organization of brain dynamics at a millisecond scale (Khanna et al., 2015; Lehmann et al., 1987; Michel and Koenig, 2018).

Recent evidence indicates that motor imagery movements modulate these parameters, with longer durations and higher occurrences of the microstate maps reflecting the coordination between sensorimotor and executive networks (Penalver-Andres et al., 2024; Zappasodi et al., 2019). This enables the investigation of how preparatory neural states are configured before actual movement execution, which may encode task-specific parameters such as load, effort, or movement intention. Building on this premise, the current study hypothesizes that EEG microstates preceding movement onset can reflect increasing load levels during a unilateral biceps curl task. The objective of the study is to identify the pre-movement microstates and their cortical sources that reflect progressively higher loads in a controlled single-joint movement, thereby elucidating the neural correlates of graded movement preparation.

## 2 Material and methods

This study recorded time-synchronized EEG, EMG, and an inertial measurement unit (IMU) while participants performed unilateral biceps curls with graded loads. Each session began with a short familiarization block, followed by experimental blocks performed. Each block consisted of ten trials and was separated by 5 min of seated rest.

### 2.1 Participants

The study was approved by the Institutional Ethics Committee, All India Institute of Medical Sciences, New Delhi (Project ID: RP04191G), and all methods were performed in accordance with the relevant guidelines and regulations. The study consisted of 11 healthy, right-handed male participants recruited from IIT Delhi (Age: mean = 23.57, ± SD = 3.2) all of whom were over 18 years old and thus considered capable of providing informed consent independently. Out of 11 participants recruited for the study, one participant was unable to complete the experimental task, thereby reducing the sample size to ten. The inclusion criteria of the study were as follows: participants having normal or corrected-to-normal vision, absence of any history of cardiovascular issues, neurological disorders, fatigue-related disorders, chronic physical conditions, and musculoskeletal diseases. Before the experiment, participants were instructed to avoid medication, tea, coffee, and alcohol for 24 h, and avoid exercise for 48 h before the experiment. To reduce the risk of selection bias, the participants were chosen randomly. Moreover, they were kept unaware of the study's objectives (to reduce performance bias) and the anticipated results (to reduce detection bias). The excluded data have been documented to prevent reporting and attention biases, respectively. All participants gave their written informed consent, and they were informed that they could leave the experiment at any time.

### 2.2. Data recording equipment

#### 2.2.1. EEG data

The study was carried out using a 32-channel saline-based EEG cap (R-net, BrainProducts GmbH, Gilching, Germany) connected to a wireless amplifier (LiveAmp-16, BrainProducts GmbH, Gilching, Germany). The Ag/AgCl electrode placement was arranged according to the international 10–10 system. For the entire acquisition, the EEG data were digitized with a sampling rate of 500 Hz, and the impedance for all electrodes was kept below 10 KΩ.

#### 2.2.2. EMG data

Time synchronized EMG activity was recorded using the Noraxon wireless Ultium EMG system, Noraxon (software - MR 3.20) at a sampling rate of 4,000 Hz. Alcohol wipes were used to clean the skin prior to electrode placement. Six surface EMG bipolar electrodes were placed over the target muscles, including the biceps brachii long and short, triceps brachii long and short, brachioradialis, and flexor carpi radialis. An adhesive tape was used to adhere the electrodes to the muscle. Electrode placement and skin preparation procedures were performed in accordance with established guidelines (Konrad, 2005).

#### 2.2.3 IMU Data

Inertial measurements were collected with a 17-IMU system (Awinda, Xsens Technologies, Enschede, The Netherlands). IMUs consist of a 3D accelerometer, a 3D gyroscope, and a 3D magnetometer. The sensors used in this study for deciphering the right biceps curl movement onset were placed over the forearm. Data from these sensors were recorded at a sampling rate of 60 Hz.

### 2.3 Experimental design

Upon arrival at the laboratory, participants were given a comprehensive briefing on the experimental protocol, and written informed consent was obtained before they participated in this study. The preparation for data acquisition involved the calibration of IMU sensors using XSens software, ensuring precise alignment. Additional preparatory measures included measuring participants' head diameters and locating the Cz point, which is determined by the intersection of the nasion-to-inion and tragus-to-tragus lines. EEG and IMU signals were recorded synchronously while participants performed unilateral (right) biceps curls (elbow flexion and extension) at varying lifted loads. IMU data from the right forearm were used to detect movement onset.

**Figure 1:**
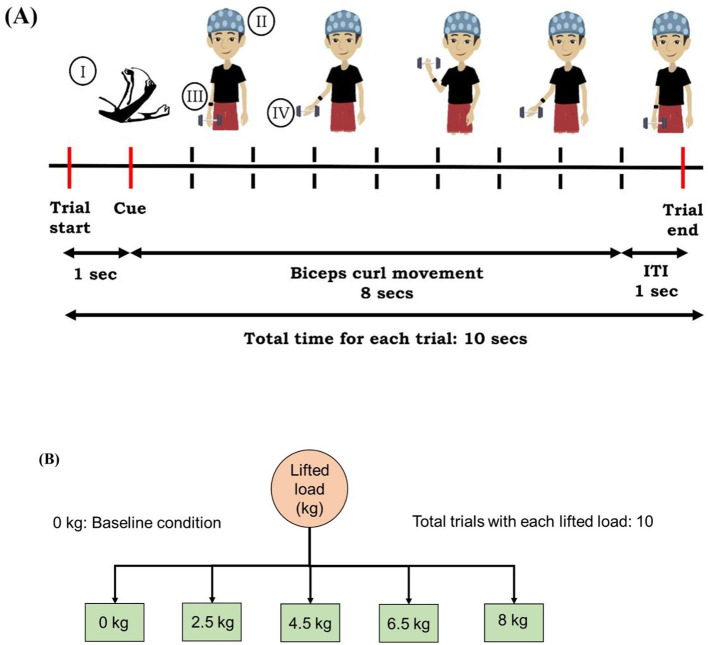
**(A)** Experimental paradigm for the unilateral right biceps curl task. Each trial began with a beep, followed by a visual cue (I) instructing the participants to initiate the movement. One complete biceps curl (elbow flexion and extension) was to be performed within 8 s. The setup included an EEG cap (II), IMU sensors (III), and lifted load (dumbbell; IV). Trials were separated by a 1 s inter trial interval. **(B)** Flowchart of increasing loads considered in the study. Each block (load condition) comprised 10 trials; 0 kg (no load) served as baseline for statistical comparisons.

Continuous EEG data were acquired from 32 channels: Fp1, Fz, F3, F7, F9, FC5, FC1, C3, T7, CP5, CP1, Pz, P3, P7, P9, O1, Oz, O2, P10, P8, P4, CP2, CP6, T8, C4, Cz, FC2, FC6, F10, F8, F4, Fp2. At the beginning of the study, participants were in a standing position with no lifted loads (dumbbells) in their hands. A monitor was positioned 3 m away in front of the participant for visual cue presentation. The experimental trials involving the right arm consisted of lifting varying loads: 0 kg (baseline), 2.5 kg, 4.5 kg, 6.5 kg, and 8 kg, with each lifted load condition. The experimental paradigm consisted of five blocks, one for each lifted load condition (0 kg—baseline, 2.5 kg, 4.5 kg, 6.5 kg, 8 kg). Participants performed 10 trials in each block. A total of 50 trials (5 blocks x 10 trials) of biceps curl were performed by the participants. At the end of each block, the experimenter manually adjusted the lifted load. The experiment was designed using PsychoPy, Open Science Tools Ltd. (version 2022.2.5) to instruct the participant to initiate the biceps-curl action. As illustrated in Figure 1, each trial started with a beep sound. 1 s after the trial start (beep), a visual cue (Figure 1A, I) was presented. Participants were instructed to perform a biceps curl after the visual cue presentation. A time period of 8 s was provided to complete the biceps curl, and the visual cue was presented throughout this period. Each trial was followed by an inter-trial interval of 1 s. A 5-min rest was given to the participants after each block to avoid muscle fatigue. The total experiment lasted approximately 30 to 40 min. The detailed experimental paradigm is shown in Figure 1.

### 2.4 Data analysis

#### 2.4.1 EEG pre-processing

**Figure 2:**
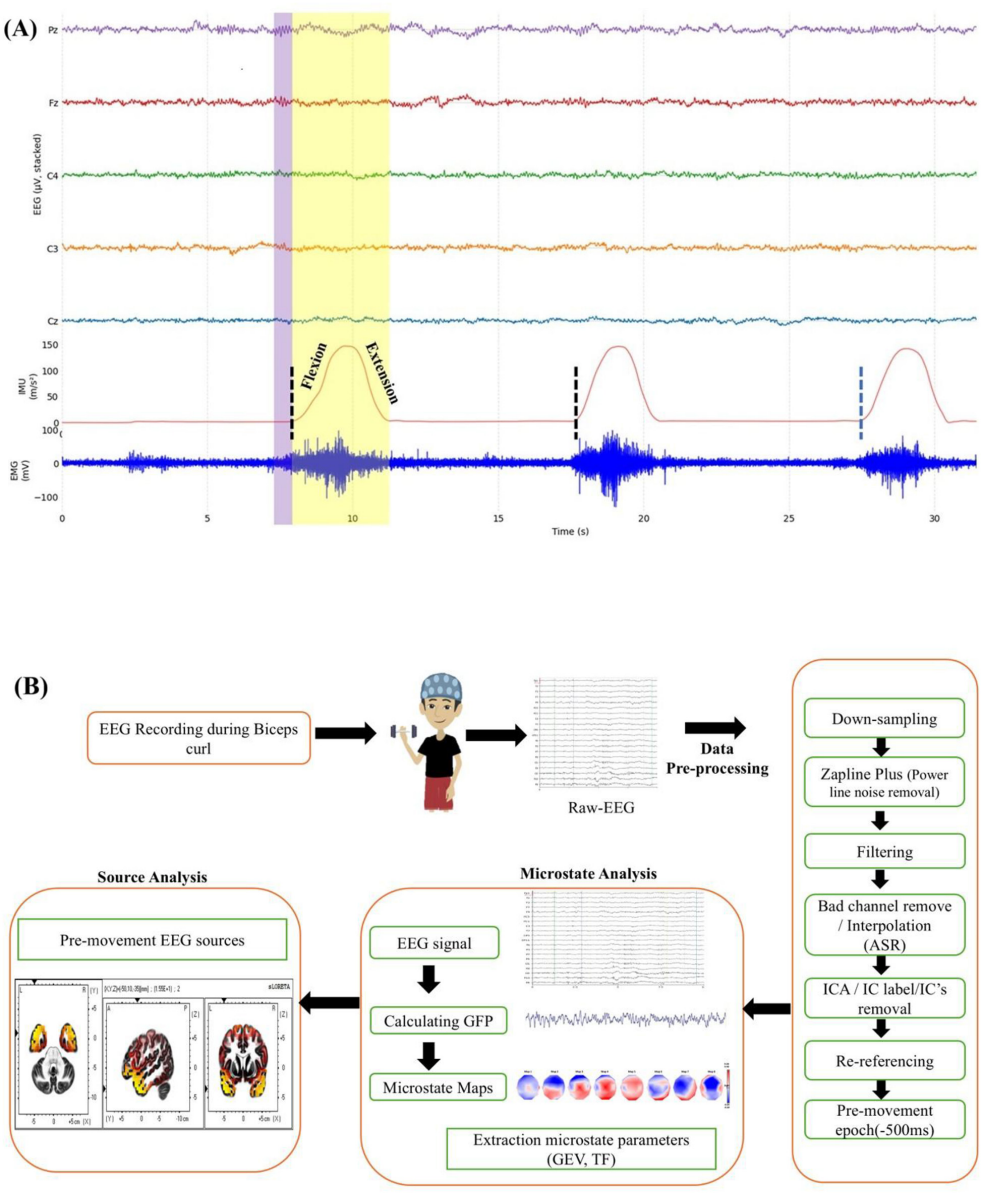
**(A)** EEG, IMU and EMG time series for unilateral biceps curls. Black dashed blue lines mark movement onsets (from IMU). Light purple window highlights pre-movement period (−500 ms to 0 s) and full movement phase is highlighted in light yellow. IMU ascending slope indicates flexion, descending slope extension. **(B)** Preprocessing and analysis pipeline for pre-movement EEG microstate analysis.

This study aimed to identify the pre-movement EEG microstates of movement intention across varying lifted loads. The movement onset was derived from IMU kinematic coordinate files representing flexion–extension of the right elbow joint and processed in MATLAB, MathWorks (version 2023b). IMU data was imported into MATLAB to mark the events corresponding to the biceps curl onset. Based on IMU-derived movement onset events, 500-ms pre-movement EEG epochs were extracted for further analysis (Figure 2A).

EEG signals were pre-processed in EEGLAB (v.2023). EEG data were downsampled to 250 Hz, and the powerline noise was removed. The data were filtered with a temporal bandpass filtering of 0.5 Hz to 30 Hz using the 4th-order Butterworth filter. The bad channels were removed (using artifact subspace rejection) with subsequent interpolation (interpolated using 3 neighboring electrodes). Independent component analysis (ICA) was applied to remove artifacts related to the eyes, muscles, heart, and channel noise from the raw signal. The cleaned signals were re-referenced to a common average reference. Baseline correction was performed for each epoch (Figure 2B).

#### 2.4.2 EMG analysis

Root-mean-square (RMS) EMG amplitude was computed for each trial and load condition to assess load-dependent changes in muscle activation. IMU data of flexion and extension data were used to segment the movement epochs for each biceps curl movement. The movement onset and offset were defined from the start of the flexion phase (rising edge) and the end of the extension phase (falling edge), respectively, and the full movement epoch was extracted (Figure 2A). Raw EMG signals were band-pass filtered at 20–450 Hz. EMG samples within each epoch were squared, averaged, and square-rooted to obtain RMS EMG. For each lifted load condition, RMS EMG were then averaged across epochs and used for the correlation analysis.

#### 2.4.3 EEG pre-movement microstate analysis

EEG microstate analyses on these EEG epochs were conducted using CARTOOL software (Brunet et al., 2011). Scalp topography maps representing pre-movement microstates were identified for each participant across all load conditions. The microstate maps corresponding to GFP peaks within the pre-movement epochs were submitted to a k-means clustering analysis to determine the most dominant and recurrent spatial configurations of the scalp electric field (Lehmann et al., 1998). This procedure extracted a set of topographic templates that characterized the pre-movement microstate classes within each subject. The optimal number of clusters (both within and across subjects), that is, the best solution representing the dominant scalp configurations, was determined using a meta-criterion approach (Bréchet et al., 2019; Custo et al., 2017; Mahini et al., 2022). Specifically, seven independent criteria were computed to evaluate clustering quality, and their individual optimal solutions were combined to obtain a single meta-criterion defined as the median of all optimal cluster numbers (Custo et al., 2017). This composite approach enhances the robustness and reliability of cluster estimation compared to earlier methods relying on a single criterion (Murray et al., 2008; Pascual-Marqui et al., 1995).

In the second step, the microstate maps best representing the pre-movement time window within each subject were identified. The spatial correlation between the subject-level template maps obtained from the first step and all pre-movement scalp topographies across conditions was computed, assigning each GFP peak to the template with the highest correspondence. The pre-movement microstates that best represented the lifted load conditions within each subject were determined based on their frequency of occurrence (number of time frames) and GEV.

In the third step, the microstate map that best represented the pre-movement time window across subjects and conditions were identified. All those maps identified in the second step were concurrently submitted to a second *k*-means cluster analysis, and its best solution was determined by means of the meta criterion.

In the fourth step, the spatial correlation was computed between the final microstate maps identified in the third step with the scalp topographic maps of the lifted load trials.

#### 2.4.4 Statistical analysis

The pre-movement microstate maps which best differentiated the increase in lifted loads were identified by statistically comparing the mean number of time frames and GEV of all the microstate maps within subjects between all the conditions by applying Friedman test followed by correction for multiple comparison using Dunn's test. All the statistical analyses were carried out using GraphPad Prism software, Dotmatics (version 6).

#### 2.4.5 EEG pre-movement source analysis

The pre-movement EEG microstate map that best differentiated the lifted load conditions (i.e., that identified in the second k-means cluster analysis) was extracted for each subject, and its intracerebral cortical sources were estimated using standardized low-resolution brain electromagnetic tomography (sLORETA) (Pascual-Marqui, 2007; Pascual-Marqui et al., 2011). Source computations were made using the Montreal Neurological Institute (MNI; Montreal, Quebec, Canada) MNI152 template (Mazziotta et al., 2001), with the solution space restricted to the cortical gray matter and hippocampi as defined by the probabilistic Talairach atlas (Lancaster et al., 2000). The intracerebral volume, i.e., sLORETA inverse solution space, was segmented into 6239 voxels of 5 mm3 each.

The difference in source localization of cortical activity between pre-movement conditions was assessed by voxel-by-voxel paired-sample *t*-tests, based on sLORETA current density values of voxels. From the resulting statistical sLORETA images, cortical voxels showing significant differences were identified by statistical nonparametric mapping (SnPM) randomizations. This randomization method (Pizzagalli et al., 2001) determined the values of the critical probability threshold for the actual observed *t-*values with a correction for multiple comparisons across all voxels. A total of 5,000 permutations were used to determine the significance of each randomization test.

## 3 Results

### 3.1 Behavioral results

#### 3.1.1 Reaction time

**Figure 3:**
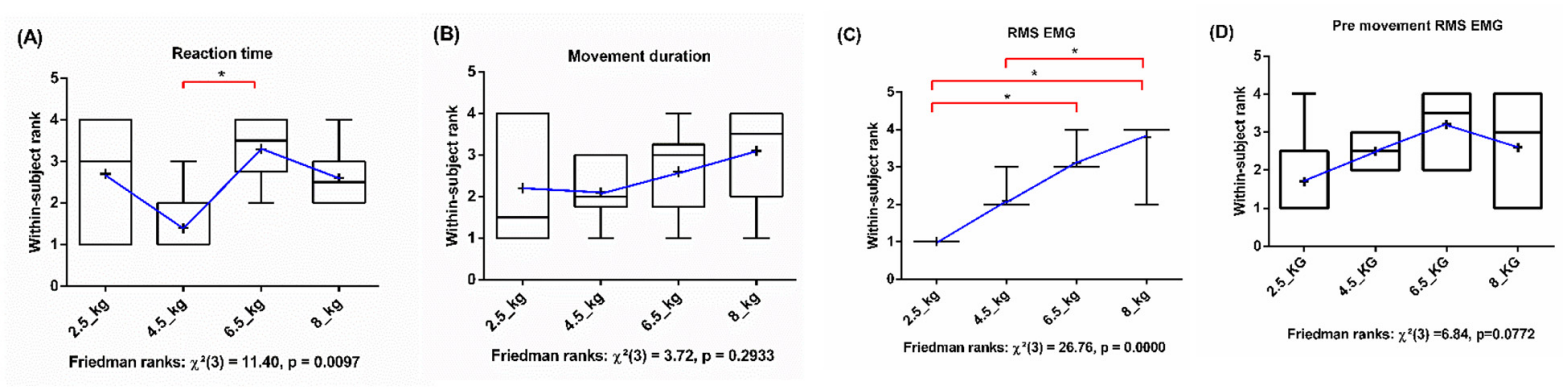
Boxplots of within-subject ranks for **(A)** reaction time, **(B)** movement duration, **(C)** RMS EMG and **(D)** pre-movement RMS EMG across loads (2.5 kg, 4.5 kg, 6.5 kg, 8 kg). Blue lines show mean ranks. Friedman's test assessed load effects; red brackets with (*) indicate significant *post-hoc* pairwise differences (*p* < 0.05). **(A)** Significant main effect of lifted load (χ^2^ (3) = 11.40, *p* = 0.0097); 4.5 kg vs. 6.5 kg. **(B)** No significant effect of varying lifted load was observed. **(C)** A statistically significant increase in rank of RMS EMG was observed when the 2.5 kg condition was compared with the 6.5 kg and 8 kg condition (*p-*value < 0.05) and 4.5kg with 8kg condition (*p-*value < 0.05). **(D)** No significant effect of pre-movement EMG for varying lifted load was observed.

The differences in within-subject reaction time across lifted load conditions (2.5, 4.5, 6.5, and 8 kg) were assessed using Friedman's rank-based analysis of variance. The study found a significant effect of load on reaction time [χ^2^_(3)_ = 11.40, *p* = 0.0097]. *Post-hoc* pairwise comparisons indicated an increase in reaction time in the 6.5 kg condition compared with the 4.5 kg condition (*p* < 0.05), (Figure 3A).

#### 3.1.2 Movement duration

Movement duration across lifted load conditions (2.5, 4.5, 6.5, and 8 kg) was assessed using Friedman's rank-based analysis of variance. The analysis did not find a statistically significant effect of load on movement duration (χ^2^_(3)_ = 3.72, *p* = 0.2933], (Figure 3B).

#### 3.1.3 RMS value of EMG

The RMS value of EMG lifted load conditions (2.5, 4.5, 6.5, and 8 kg) were assessed using Friedman's rank-based analysis of variance. A significant effect of lifted load was observed on muscle activity (χ^2^_(3)_ = 26.76, *p* = 0.0000), indicating that muscle recruitment increased as the lifted load increased. *Post-hoc* pairwise comparisons revealed significant increases in RMS EMG ranks in the 6.5 kg and 8 kg conditions compared with the 2.5 kg condition, as well as from the 8 kg condition compared with the 4.5 kg condition (*p* < 0.05), (Figure 3C). The RMS value of pre-movement EMG for the lifted load conditions (2.5, 4.5, 6.5, and 8 kg) did not show any significant difference (Figure 3D).

### 3.2 Pre-movement EEG microstates

The pre-movement template maps that best represented pre-movement microstates between conditions within each lifted load condition were identified. The meta-criterion in the k-means cluster analysis identified template maps as the optimal solution with average (SD) values of 9.4 (0.54), which explained the global variance with average (SD) values of 78.11% (10.39%).

**Figure 4:**
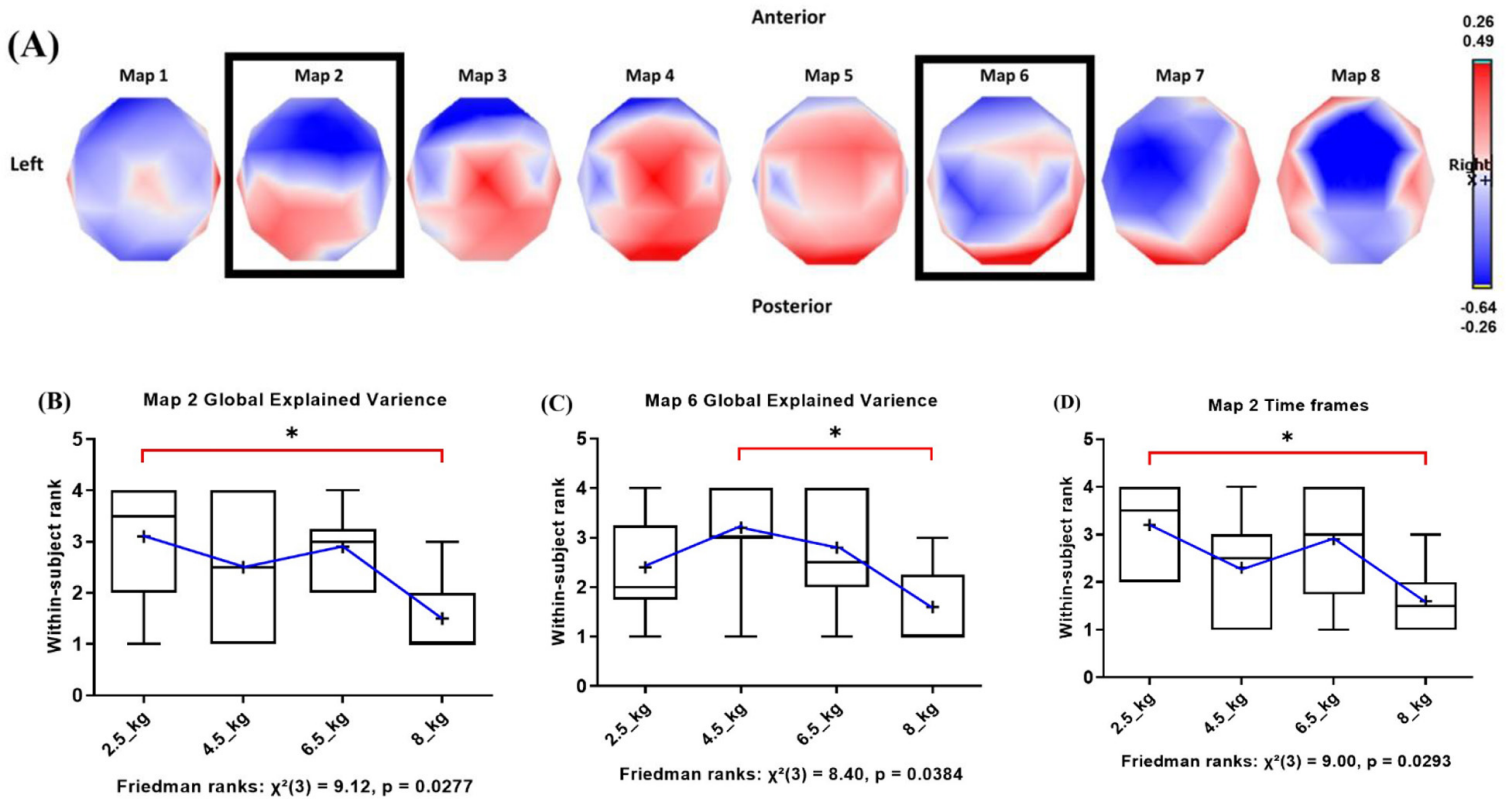
**(A)** Eight scalp topographies of pre-movement microstate maps (identified by k-means clustering; −500 ms of movement onset). Maps 2 and 6 (highlighted) showed statistically significant differences between lifted loads on microstate parameters. Color scale: red (positive), blue (negative) potentials. Within-subject declining ranks of **(B)** Map 2 GEV (2.5 kg vs. 8 kg), **(C)** Map 6 GEV (4.5 kg vs. 8 kg), **(D)** Map 2 time frames (2.5 kg vs. 8 kg) were observed across loads. Red brackets and asterisk (*) indicate significant *post-hoc* differences (*p* < 0.05).

The meta criterion in the second k-means cluster analysis identified 8 pre-movement template maps as the optimal solution across lifted loads conditions (Figure 4). These 8 maps explained 69.07% of the variance.

Two pre-movement maps (Maps 2 and 6) were found to differentiate the lifted loads. Map 2 showed significant differences in GEV and the number of time frames (*p* < 0.05), and Map 6 also showed a significant difference in GEV (*p* < 0.05).

### 3.3 Pre-movement EEG sources

**Figure 5:**
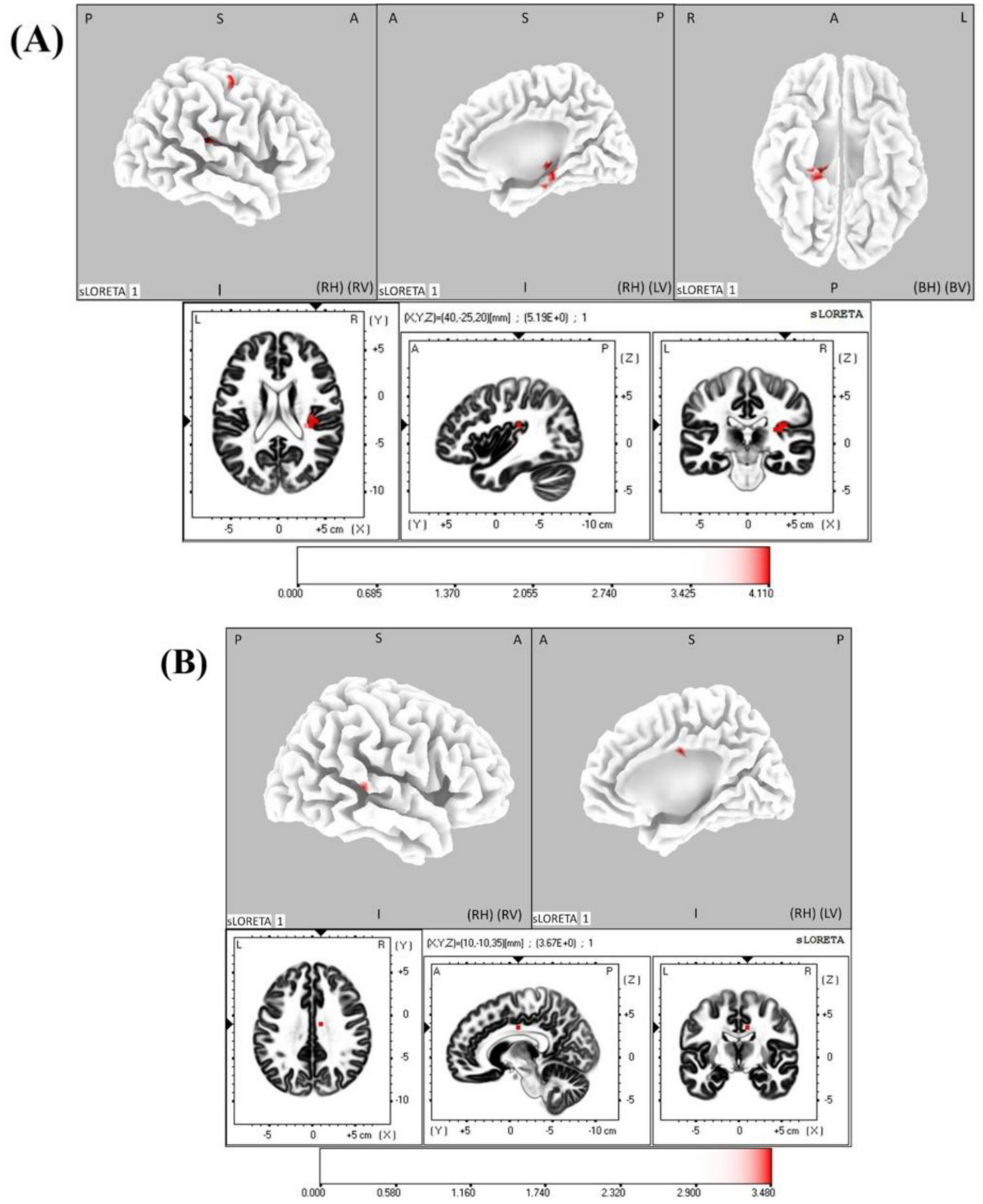
sLORETA statistical maps of pre-movement source differences for **(A)** microstate Map 2 and **(B)** Map 6. Colored regions show voxels with significant current source density differences (*p* < 0.05). Significant clusters are displayed on a fiducial cortical surface **(top)**, MRI template **(middle)**, and voxels of highest statistical significance: right insula (Map 2; *t* = 4.054) and right cingulate gyrus (Map 6; *t* = 3.465). Orientation: L/R (left/right), A/P (anterior/posterior), S/I (superior/inferior).

Statistical analyses on sLORETA images for the pre-movement EEG microstates (Maps 2 and 6) that differentiated the lifted load conditions and showed increased activations immediately preceding movement are shown in Figure 5. In map 2, the source maxima were localized at right insula (MNI coordinates of the maxima: 40, −25, 20, *t* = 4.054, *p* < 0.05). Besides source maxima, supra-threshold cortical voxels that showed increased activations just prior to movement were found localized at the parahippocampal gyrus and the precentral gyrus (Table 1). In map 6, the source maxima were localized at right cingulate gyrus (MNI coordinates of the maxima: 10, −10, 35, *t* = 3.465, *p* < 0.05). In addition to right cingulate gyrus, supra-threshold cortical voxels that showed increased activations were found localized at the superior temporal gyrus (Table 1).

**Table 1:** Intracranial generators of premovement microstate maps that differentiated lifted load conditions.

**Microstate map 2 sources**	
Brain region	Brodmann area
Insula	13 (R)
Parahippocampal gyrus	27, 28, 35 (R)
Precentral gyrus	6 (R)
**Microstate map 6 sources**	
Brain region	Brodmann area
Cingulate gyrus	24 (R)
Superior temporal gyrus	42 (R)

R- Right hemisphere.

### 3.4 Correlation results

**Figure 6:**
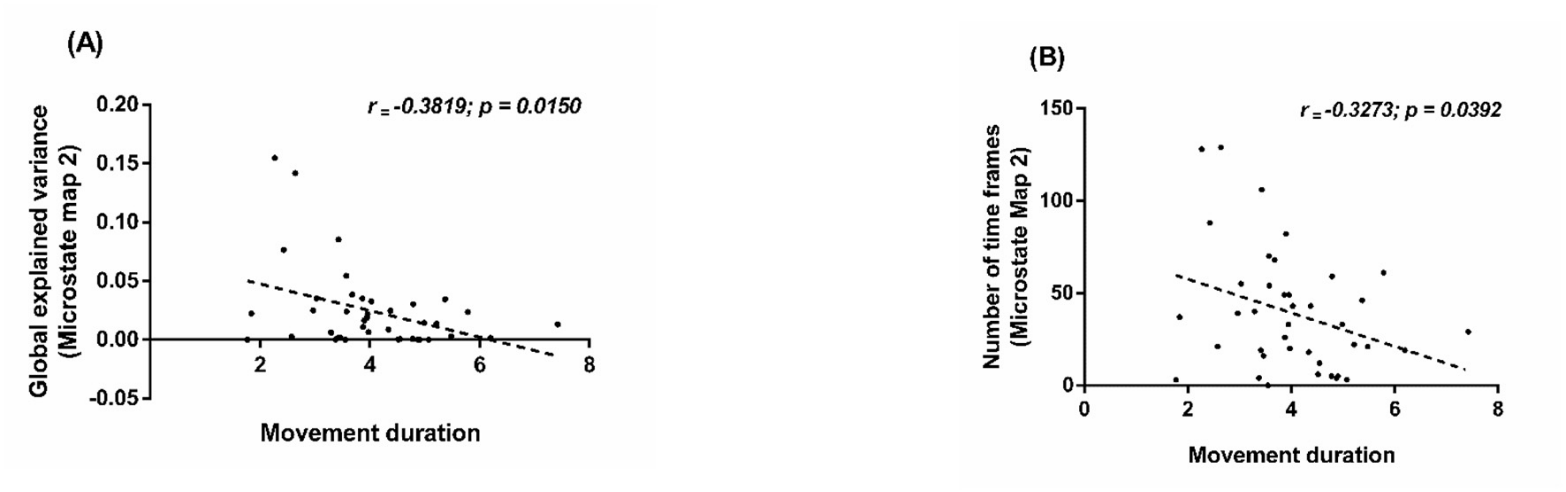
Scatterplots of associations across loads (2.5, 4.5, 6.5, 8 kg) between **(A)** movement duration and Map 2 GEV, **(B)** movement duration and Map 2 time frames. Dots are individual data points; dashed lines show the linear fits. Pearson's *r* and *p-*values (on plots) indicate correlation strength and significance.

Pearson's correlation analysis, performed to assess associations between the pre-movement microstate maps that showed significant difference with increasing lifted loads (Map 2 and Map 6) and performance metrics, including reaction time (RT), movement duration (MD), and RMS EMG. Movement duration negatively correlated with the GEV (*r* = −0.3819, *p* = 0.0150; Figure 6A) and number of TF of microstate map 2 (*r* = −0.3273, *p* = 0.0392; Figure 6B). Reaction time and RMS EMG did not show significant correlations with pre-movement microstate map 2 measures (TF, GEV). We did not find any significant correlations between pre-movement microstate map 6 measures (TF, GEV) and performance metrics (RT, MD, RMS EMG).

## 4 Discussion

The present study investigated changes in large-scale network dynamics using EEG microstates analyses during the pre-movement phase under increasing lifted load conditions. Out of eight pre-movement EEG microstate maps, two maps (Maps 2 and 6) were markers of increased lifted load conditions. Specifically, the pre-movement GEV and time frames differentiated between the 2.5 kg, 4.5 kg, and 8 kg lifted load conditions.

The pre-movement microstates were measured within the 500 ms interval preceding the movement onset to capture the dynamics of “prepared” movement as suggested by readiness potential (RP) and ERD of sensorimotor rhythms (Ghosh et al., 2025) and to focus on the critical juncture around 200 ms where the brain commits to movement execution, a “point of no return” (Schultze-Kraft et al., 2016). While most studies focused on source activations during motor imagery (MI) and movement execution (ME) (Choi and Choi, 2022; Yao et al., 2025), EEG microstates measures changes in large-scale network dynamics. Resting-state EEG microstate dynamics predict MI-BCI performance and cue-guided MI elicits microstates similar to motor execution (Cui et al., 2023; Wang et al., 2023). EEG microstate parameters differentiate right vs. left hand movement preparation (Zhang et al., 2022). To the best of our knowledge, this is the first study demonstrating that pre-movement EEG microstates reflect the encoding of graded lifted load information before movement onset.

### 4.1 Pre-movement microstate map 2—microstate measures, associates sources, and correlation

In the present study, pre-movement map 2 which resembled clinical microstate class B (right anterior—left posterior) appeared for a lesser number of times and had lower GEV when the lifted loads increased (Valt et al., 2024). A reduction in frequency of occurrence of EEG microstates during motor preparation (L. Zhang et al., 2022), a decrease in the duration and time coverage of this microstate with increasing grip force of both executed and imagined movements (Fu et al., 2018), and a reduction in GEV during higher mental work load that reflects attention concentration (Li et al., 2025; Jia et al., 2021) have been reported. In the present study, the decrease in microstate map 2 parameters observed during the premovement preparation phase may reflect the shift from internally oriented to externally directed attention processing as task demands rise with increasing lifted load.

Insula showed the dominant activity, with additional source activations in the parahippocampal gyrus and precentral gyrus while lifted load increased. Insula has been shown to be activated gradually with muscle pain during sustained effort, highlighting its role in processing interoceptive and fatigue-related signals (Spring et al., 2017). Insula has fundamental role in interoceptive awareness i.e., subjective sense of the physiological condition of the entire body, and somatosensory processing such as touch, kinesthesia and proprioception (Tinaz et al., 2018). Previous studies highlighted that simple movements may not always trigger the insula, and its activation emerges during increase in metabolic rate or attention-intensive actions especially when movements require increased effort (Blinkenberg et al., 1996; Weiller et al., 1996). Increase in the muscle activity (RMS) and movement duration when lifted load became heavier are often associated with slower responses and reduced motor readiness (Li et al., 2018; Wolkorte et al., 2014). Likewise, in the current study, the reaction time varied across load conditions, with a significant increase between the 4.5 kg and 6.5 kg loads. Integrating effort related interoceptive signals and movement preparation is one of the primary functions of the insula (Tinaz et al., 2018; Williamson et al., 2006; Wang et al., 2019). In light of these reports, pre-movement insular engagement through microstate map 2 and its negative correlation with movement duration in the current study indicate the pivotal role of insula in movement preparation during increasing physical demands on subsequent movement execution.

Parahippocampal gyrus is increasingly recognized for its role in predictive action processing (Stadler et al., 2011), as well as activation during pre-movement and action-observation (Calvo-Merino et al., 2005). The increased activation under higher lifted loads suggests that parahippocampal gyrus integrates contextual information derived from prior experience of lifted load, thereby anticipating the postural adjustments and level of effort required for the lift.

Alongside right insula and right parahippocampal gyrus, intracranial generators of premovement microstate map 2 was localized at right precentral gyrus. Precentral gyrus, is responsible for executing voluntary movements and exhibit motor-related activity approximately 2 s before movement onset (Cohen et al., 2010; Bookheimer, 2013). The precentral gyrus exhibits robust pre-movement activity encoding direction force, and amplitude of the intended action (Vargas-Irwin et al., 2025). Increased activation of the precentral gyrus with increase in lifted load in our study reflects enhanced motor preparation for scaling the required force prior to movement execution.

The present findings suggest that the reduced GEV and time frames of premovement microstate map 2 reflect large-scale neural dynamics integrating effort-related interoceptive signals with anticipatory motor control as physical demands increase. Premovement source activation in the right insula, right parahippocampal gyrus, and precentral gyrus is associated with interoceptive signal integration, postural adjustments, and required force scaling, respectively.

### 4.2 Pre-movement microstate map 6—microstate measures and associated sources

Pre-movement map 6 in the present study resembled canonical microstate class A (left anterior-right posterior orientation), which has been related to auditory processing and cognition within large scale cortical networks (Aeschlimann et al., 2025; Michel and Koenig, 2018). Notably, only the GEV of this map decreased with increase in lifted load, with the lowest GEV at the heaviest weight, whereas its temporal parameter (number of time frames) remained stable across conditions.

Hao et al. compared resting state EEG microstates between healthy controls and stroke survivors. Results showed that microstate class A exhibited significantly lower GEV in healthy controls at rest compared with stroke patients (Hao et al., 2022). Converging evidence suggests that microstate class A is sensitive to task engagement and is related to participants' arousal rather than being exclusively a “resting” pattern. In a simulated flight study, Han et al. reported that microstate class A parameters (duration and occurrence) were suppressed with high mental fatigue under high attention demands (Han et al., 2024). In a psychomotor vigilance task, Di Muccio et al. found that individuals with higher cardiorespiratory fitness displayed a lower prevalence of microstate class A during pre-stimulus periods, which the authors interpreted to reflect an increased vigilance state (Di Muccio et al., 2023). Wang and Li reported that microstate class A dominated during arm raising motor imagery task, showing the highest GEV during lateral lowering of arms (Wang and Li, 2025). Based on these varied roles indicated by the previous studies, pre-movement reduction in GEV of microstate class A (map 6) with increase in physical demands in this study could reflect increase in vigilance and task engagement.

Intracranial generators for Map 6 were localized at the right cingulate gyrus and superior temporal gyrus showing significantly higher activation in higher lifted load condition. This observation is consistent with a previous finding that the cingulate gyrus, together with the medial prefrontal cortex, insula, and thalamus, is involved in processing the perceived sense of effort during physically demanding tasks (Williamson et al., 2006). The cingulate cortex and supplementary motor area are strongly interconnected with the insula and together support motor control, action selection, and performance monitoring (Tinaz et al., 2018). In light of these evidence, increased activation of the cingulate gyrus with higher lifted loads in the present study likely reflects increased evaluation of internal physiological signals related to effort when the physical demands increase.

The planning and execution of voluntary movement requires that the brain extracts sensory information regarding body position and envisages future positions, by integrating multisensory inputs with ongoing and planned motor activity. Neuroimaging studies have referred the superior temporal gyrus as the multimodal integration center and this region is recruited when upper-limb actions require enhanced monitoring of movement and body state. In accordance with this evidence, the superior temporal gyrus showed higher activation when the participants lifted heavier loads. During arm tasks post stroke, increased activation of the superior temporal gyrus has been reported alongside motor and parietal regions (Bolognini et al., 2016). Similarly, superior temporal areas has been studied to be engaged during execution and observation of goal-directed reaching and grasping, particularly when movement kinematics place greater demands on sensory integration (Gazzola and Keysers, 2009).

Therefore, the premovement Map 6 exhibited a load-dependent reduction in GEV, consistent with heightened arousal and increased task engagement under greater physical demand. Source activation in the cingulate and superior temporal gyrus suggests increased effort monitoring and sensory integration during motor preparation.

The limitation of the present study is its relatively small sample size (*n* = 10), which affects the generalizability of the findings. Future work should examine whether these pre-movement microstates can be decoded in real time for closed-loop applications with a larger sample size. The current study employed a graded increase in lifted load conditions, which may have induced fatigue and time-on-task effects, potentially confounding the study results. Future studies on randomized lifted load paradigm are required to validate the study findings.

## 5 Conclusion

Brain regions implicated in interoception, postural control, performance monitoring, multisensory integration and force regulation, indicating stronger recruitment of motor preparatory brain areas before movement onset with increasing load was demonstrated in the study. Two EEG microstate maps parameters could be used to reflect the preparedness of the networks for higher lifted load before movement onset. This differentiation in EEG microstates offers novel insight into how the brain adapts preparatory neural networks in response to increasing motor demands. This study suggests the possibility of creating EEG microstates-based control systems for BCI of assistive devices for predicting the required force in real-time that can help improve the performance of such systems already in existence.

## Data Availability

The raw data supporting the conclusions of this article will be made available by the authors, without undue reservation.
